# Uncommon cause of radiculopathy: A case of symptomatic Tarlov cyst in an elderly female and literature review

**DOI:** 10.1002/ccr3.9189

**Published:** 2024-07-16

**Authors:** Shritik Devkota, Sugat Adhikari, Samiksha Lamichhane, Bishal Koirala, Arif Hussain Sarmast

**Affiliations:** ^1^ Department of Radiodiagnosis and Imaging Anil Baghi Hospital Punjab India; ^2^ Shreegaun Primary Health Care Center Dang Nepal; ^3^ Department of Radiodiagnosis and Imaging B. P. Koirala Institute of Health Sciences Dharan Nepal; ^4^ Department of Neurosurgery Anil Baghi Hospital Punjab India

**Keywords:** neuropathic pain, radiculopathy, sacral spinal cysts, Tarlov cysts, Type II spinal meningeal cysts

## Abstract

**Key Clinical Message:**

Tarlov cysts are uncommon causes of sacral radiculopathy, with particular predilection to second and third sacral roots, requiring timely diagnosis with lumbosacral MRI, and surgical management if symptomatic.

**Abstract:**

Tarlov cysts or Type II meningeal cysts, are CSF‐filled sacs located in the extradural space of the sacral spinal canal, commonly originating at the dorsal root ganglion. While they were first documented by Tarlov in 1938, their etiology remains uncertain, with theories suggesting trauma‐induced bleeding or congenital abnormalities. These cysts, estimated to affect between 1% and 9% of the adult population, typically manifest as incidental findings but may lead to symptoms such as radiculopathies, sacral pain, and weakness in related sacral muscles. We present a case of a 63‐year‐old female presenting with recurrent left buttock pain and radiating leg discomfort. Physical examination revealed tenderness in the left buttock region, positive straight leg raise test, and minimal sensory deficits in the S1‐S2 dermatomes. A provisional diagnosis of radiculopathy was made, prompting further evaluation with MRI, revealing a Tarlov cyst and absence of lumbar spinal canal stenosis or neural foraminal compromise. The patient declined intervention and was managed conservatively. This case highlights the diagnostic challenges and therapeutic considerations in managing symptomatic Tarlov cysts, emphasizing the importance of tailored treatment strategies.

## 1 INTRODUCTION

Tarlov cysts are CSF‐filled sacs situated in the extradural space of the sacral spinal canal, forming within the nerve root sheath at the dorsal root ganglion. They were first reported by Tarlov in 1938 as incidental findings at autopsy, subsequently classified as Type II meningeal cysts.^1^, ^2^ Tarlov cysts typically develop at the junction of the posterior root and the dorsal ganglion, positioned between the perineurium and endoneurium. The definition of a Tarlov cyst is histopathological, requiring the presence of spinal nerve root fibers in the cyst wall or its cavity.^2^ Prevalence of these lesions have been estimated to affect between 1% and 9% of the adult population.^2^, ^3^, ^4^, ^5^ The prevalence of TCs is 1.5%–4.6% of which 22% were symptomatic with a female preponderance.^6^ A marked predilection toward TCs among female patients was evident, with a female‐to‐male ratio of 3:1, compared to 1.4:1 in the overall study cohort. This results in a pronounced female preponderance, constituting 67.7% of TC cases. Moreover, TC prevalence increases with advancing age, strengthening the proposition that these lesions are primarily acquired rather than congenital in nature.^7^ Tarlov cysts are uncommon and underdiagnosed, with notable complexities in diagnosis and management.

## 2 CASE REPORT

### 2.1 Case history and examination

A 63‐year‐old female, presented to the ER with a chief complaint of recurrent mild pain in her lower back and left buttock, with radiation down her left lower limb. She reported that the pain had been present for more than 6 months and was progressively worsening, impacting her ability to walk and perform daily activities. Upon further inquiry, she described the pain as sharp and shooting, exacerbated by movement. She also noted occasional numbness and tingling sensations in her left lower limb, particularly in the toes. These symptoms worsened while coughing, standing, or climbing up and down the stairs. She had been taking metformin and amlodipine‐losartan for diabetes mellitus and hypertension regularly. Other than that, her past history was insignificant. There was no bowel or bladder dysfunction.

During the physical examination, tenderness was noted upon palpation of the left buttock region, with no signs of inflammation or skin abnormalities. Straight leg raise test was positive on the left side, reproducing the patient's symptoms of pain radiating down the leg. Minimal sensory deficits were noted in the region of the S1 and S2 dermatome, while motor functions remained intact with power in all limbs noted 5/5. Ankle jerk reflex was slightly reduced on the left side.

### 2.2 Diagnostic evaluation

**FIGURE 1:**
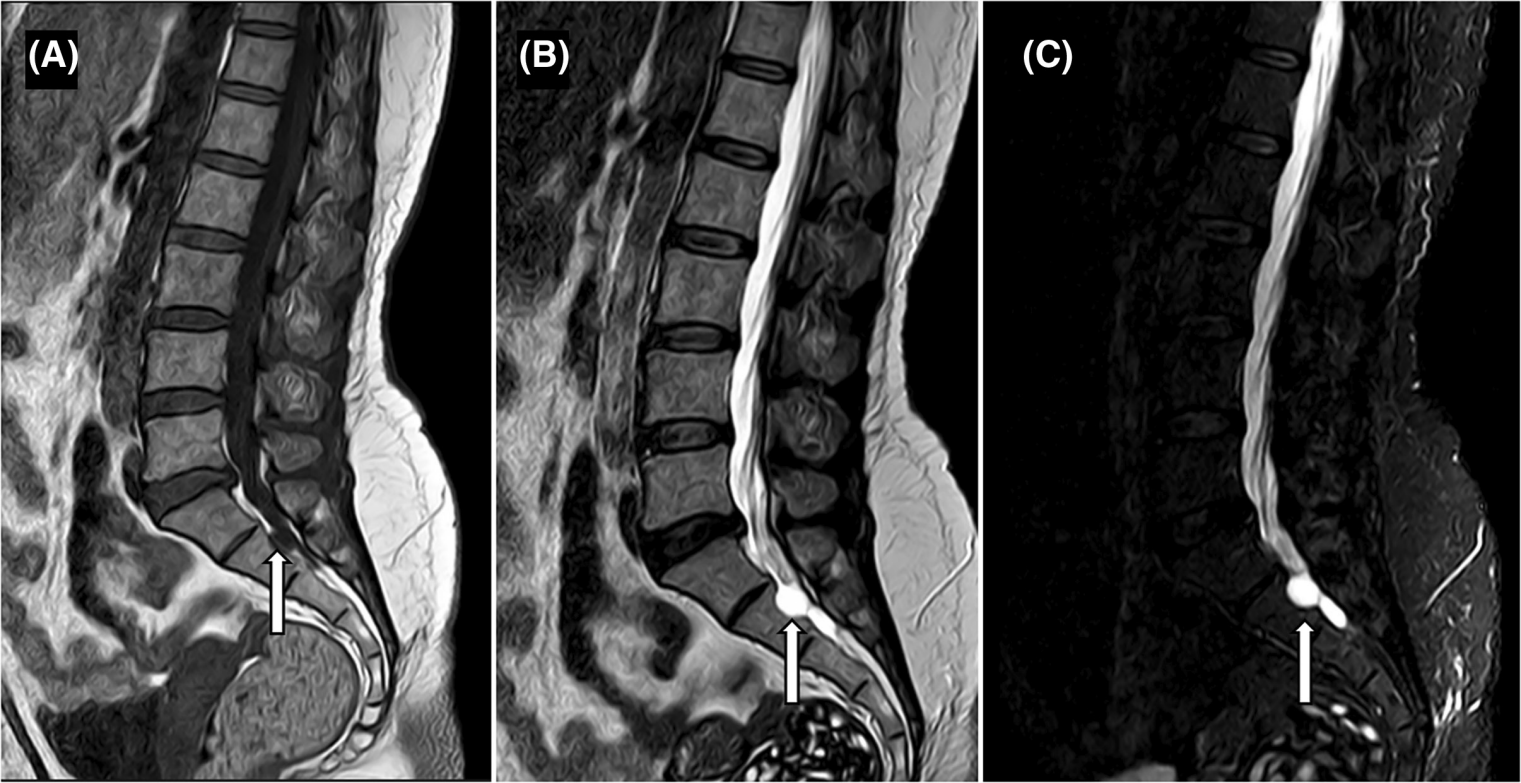
Sagittal T1W (A), T2W (B) and STIR (C) sequences showing T1 hypointense and T2/STIR hyperintense cyst (white arrows) causing mild scalloping of S2 vertebra along its neural foramen. No spinal canal stenosis or neural foraminal compromise was noted at the level of lumbar spine.

**FIGURE 2:**
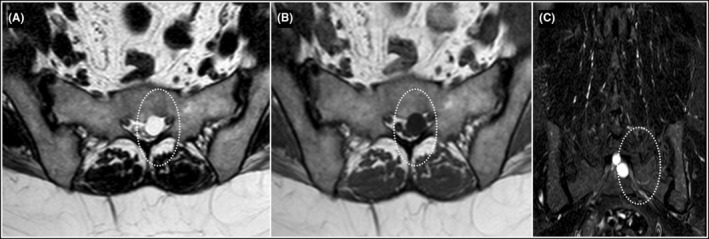
Axial T2W (A), T1W (B), and Coronal STIR (C) Images show cyst (dotted white circle) along the left neural foramen causing scalloping and effacement of the anterior aspect of the left neural foramen with compression of left exiting nerve roots at this level.

Based on the clinical presentation and physical examination findings, a provisional diagnosis of radiculopathy was made, indicating compression or irritation of the nerve roots in the lumbar spine at the level of S2. Further diagnostic evaluation, with MRI scan, was recommended to identify the underlying cause of the radiculopathy and determine the appropriate course of treatment. MRI showed T1 hypointense, T2/STIR hyperintense cystic lesion of size approximately 10 mm × 11 mm × 12 mm (anteroposterior × transverse × craniocaudal dimensions) along left neural foramen at S1‐S2 level causing scalloping and effacement of anterior aspect of left neural foramen with narrowing of left exiting nerve roots (Figures 1 and 2). No spinal canal stenosis or nerve root compression were seen at lumbar spine. Therefore, left sided Tarlov cysts emerged as a possible source of the patient's radiculopathy. Patient was explained about both interventional and conservative management.

### 2.3 Management and follow‐up

Following a discussion of both surgical and non‐surgical treatment options, the patient declined surgery and opted for conservative management with NSAIDs and pregabalin. Exercises for abdominal muscle strengthening, McKenzie exercises for relieving back pain, and hamstring stretching were recommended for which the patient reported good compliance. At 3 months follow‐up, she reported having significantly reduced symptoms and had improved functionality.

## 3 DISCUSSION

Spinal meningeal cysts are categorized into three types: Type I, which are extradural cysts without spinal nerve root fibers; Type II, known as Tarlov cysts, which are extradural cysts with spinal nerve root fibers; and Type III, which are intradural meningeal cysts.^4^ Tarlov Cysts, filled with cerebrospinal fluid (CSF), typically maintain a constant size and are commonly found at the posterior sacral or coccygeal nerve roots, with a predilection for the second or third sacral roots.^3^, ^4^, ^5^, ^6^, ^7^

The exact cause of these cysts is still uncertain. One theory suggests they may result from trauma, leading to bleeding in the subarachnoid space. This bleeding could block the drainage of veins in the perineurium and epineurium, causing them to rupture and form cysts. Another theory proposes a congenital origin, where abnormal growths in the root sleeve obstruct normal CSF flow. The formation of cysts may also involve a “ball valve” mechanism, where CSF flows into the cyst but is restricted from flowing out, causing it to expand. Histological examination typically shows an outer wall of vascular connective tissue and an inner wall lined with flattened arachnoid cells, sometimes containing nerve fibers and ganglion cells.^3^

Tarlov cysts have been associated with various symptoms, including coccygodynia, sacral pain, radiculopathies, sacral insufficiency fractures, weakness in related sacral muscles, and bowel and bladder issues. Specific radicular pain may result from nerve root distortion, compression, or stretching by the cyst. Symptoms may worsen with posture changes, coughing, valsalva maneuvers, standing, lifting, or climbing stairs, which increase CSF pressure, but can be alleviated by lying down.^3^, ^8^ Approximately only below 1% of sacral perineural cysts become large and cause symptoms related to local compression.^8^ There was no lumbar spinal canal stenosis or lumbar neural foramen compromise to explain the nature of patient's clinical presentation. Our patient's symptom could be explained by local sacral nerve roots compression caused by the Tarlov cysts. Tarlov cysts are symptomatic in 20% of patients.^9^

Lumbosacral MRI is considered the imaging study of choice in identifying tarlov cysts because these cysts are filled with CSF, a low signal is seen on T1 and a high signal is noted on T2. MRI in our case revealed an 8.5 mm × 11 mm × 12 mm cystic lesion at the S1‐S2 level, causing compression of the left neural foramen and narrowing of the left exiting nerve roots. The majority of Tarlov cysts are incidental findings on MRI.^5^

When MRI is unavailable, CT myelogram serves as an alternative for detecting perineurial cysts. CT scans, with or without intrathecal contrast, reveal these cysts as isodense with CSF, often causing bone abnormalities. Post myelography CT scans effectively show cyst communication with the spinal subarachnoid space and surrounding bone scalloping. Compared to CT, MRI offers superior tissue resolution, multiplanar views, and noninvasiveness. Myelography with water‐soluble contrast fills the cyst more rapidly than oil‐based contrast. Plain x‐rays may display spinal canal or sacral foramina erosions. However, while imaging aids diagnosis, confirming a Tarlov cyst requires histopathological examination to differentiate it from similar spinal conditions.^2^, ^3^, ^4^

It is generally agreed that asymptomatic Tarlov cysts do not require treatment.^4^ Treatment options for sacral perineural cysts include conservative and surgical approaches. Conservative treatment involves pain medication, physical therapy focusing on McKenzie exercises, pelvic stabilizer and abdominal strengthening, and hamstring stretching. Due to the rarity and unclear nature of these symptomatic cysts, there is no consensus on the best treatment. Patients with radicular pain initially undergo medical treatment with anti‐inflammatory drugs and physical therapy. All of these conservative measures were advised to our patient with a fairly good reported outcome, despite the cyst being symptomatic.

Surgery is considered if conservative measures fail, particularly for patients with cysts larger than 1.5 cm, along with radicular pain or bowel/bladder dysfunction.^10^, ^11^, ^12^ Neurosurgical procedures for symptomatic Tarlov cyst(s) include more radical techniques like simple decompressive laminectomy, lumbo‐peritoneal shunting, and cyst‐to‐subarachnoid shunt placement. Advanced microsurgical methods involve cyst fenestration (partial sacral laminectomy and fenestration of the cyst, with fibrin glue injection into the cyst lumen), sealing the introitus and communicating channels with muscle grafts, and nerve root imbrication (sacral laminoplasty, nerve root imbrication, and wrapping with a collagen matrix (DuraGen™), sometimes with fibrin glue reinforcement of the reconstructed nerve root). Immediate and short‐term postoperative complications in the fenestration and imbrication groups contained CSF leak or “pseudomeningocele,” wound infection, wound dehiscence, chemical meningitis, and treatment failure.^9^, ^11^ The postoperative complication rate in patients undergoing surgical intervention was 16.9% (11.8–22.7) and cyst recurrence was 8.5% (3.5–15.4).When a complication occurred, the most frequent complication of surgical intervention was the development of a surgical site infection and/or CSF leak.^6^ Electrophysiological monitoring helps reduce damage to sacral nerve roots. Follow‐up typically includes MRI scans at 6 months and 1 year postoperatively.^3^, ^4^, ^8^

## 4 CONCLUSION

Despite being an extremely rare cause of radiculopathy in elderly females, Type II spinal meningeal cysts also known as Tarlov cysts can sometimes cause significant morbidity in patients. Diagnostic difficulties might lead to challenges in management but these cysts should always be considered when mass of cystic nature is seen especially in the region of second or third sacral roots. Lumbosacral MRI is the gold standard imaging modality of choice for diagnosis of Tarlov cysts. Conservative management is preferred for asymptomatic cases, whereas surgical management should be done for large cysts causing significant symptoms. Limitations encountered during the management of our case included slight delay in the diagnosis due to late presentation of the patient despite 6 months of symptoms, and the refusal of the patient for surgical procedure, which left conservative management as the only management option.

## AUTHOR CONTRIBUTIONS

**Shritik Devkota:** Conceptualization; data curation; writing – original draft; writing – review and editing. **Sugat Adhikari:** Conceptualization; data curation; writing – original draft; writing – review and editing. **Samiksha Lamichhane:** Data curation; writing – original draft; writing – review and editing. **Bishal Koirala:** Writing – original draft; writing – review and editing. **Arif Hussain Sarmast:** Conceptualization; data curation; supervision; writing – original draft; writing – review and editing.

## FUNDING INFORMATION

No fund was available for this study.

## CONFLICT OF INTEREST STATEMENT

The authors declare no conflict of interest in this study.

### ETHICS STATEMENT

The patient has provided written informed consent for the publication of this case report.

### CONSENT

Written informed consent was obtained from the patient to publish this report in accordance with the journal's patient consent policy.

## Data Availability

Data sharing is not applicable to this article as no new data were created or analyzed.
